# 

**Published:** 1891-03

**Authors:** 


### CONTENTS FOR MARCH, 1891.

ORIGINAL COMMUNICATIONS.

Mechanical Restraint in our State Hospitals for the Insane. By Gros. R. Trowbridge, A. M., M. D. .... 449
Ovarian and Ligamentous Cysts Co-existing in the Same Patient; Operation; Death from Shock. By William Warren Potter, M. D. .... 457

SOCIETY PROCEEDINGS.

Medical Society of the County of Erie .... 459
Gynecological and Obstetrical Society of Baltimore .... 471

CLINICAL REPORT.

Surgical Cases Treated at the Hospital of the Sisters of Charity .... 478

PROGRESS IN MEDICAL SCIENCE.

Ophthalmology .... 484
Neurology .... 488
Therapeutic Notes .... 489

SELECTION.

Some Notes Bearing on the Administration of Iron .... 492

EDITORIAL.

Medical Society of the State of New York .... 497

PERSONAL.

Dr. L. S. McMurtry — Dr. Charles A. L. Reed .... 501

SOCIETY NOTES AND MEETINGS.

The American Electro-Therapeutic Association .... 501
Dr. Benjamin Lee — The Buffalo Obstetrical Society — The American Medical Association — National Meeting of Railway Surgeons .... 502

JOURNALISTIC NOTES.

The *Neurologisches Centralblatt* — The Microscope .... 502

*Anales de la Asistencia Publica* — *Revista de Ciencias Medicas de Barcelona* .... 50

BOOK REVIEWS.

A Text-Book of Comparative Physiology .... 503
An Illustrated Encyclopedic Medical Dictionary .... 504
A Practical Treatise on Headache, etc .... 505
Ointments and Oleates — Operation Blanks .... 506
Physical Diagnosis and Practical Urinalysis .... 507
Transactions of the Medical and Chirurgical Faculty of the State of Maryland .... 508
Modern Treatment of Headache — Post-Mortems .... 508
Books and Pamphlets Received .... 509

MISCELLANY.

Vick's Floral Guide for 1891 .... 511
Recent Medical Patents — The American Association of Obstetricians and Gynecologists 512

